# Supplementary material for: The role of MHC supertypes in promoting trans-species polymorphism remains an open question
Source: Nat Commun. 2018 Oct 19;9:4362. doi: 10.1038/s41467-018-06821-x (PMC6195607; doi:10.1038/s41467-018-06821-x)
Supplement: Supplementary file 1 — Supplementary Information [file 41467_2018_6821_MOESM1_ESM.pdf]

Supplementary Information

The role of MHC supertypes in promoting trans-species polymorphism remains unsupported

Ejsmond et al.

Supplementary Figure 1. Effects of delete-one jack-knifing of each of Lighten et al.'s [1] 15 MHC supertypes (STs) on ST-based population genetic structure. In each plot, red dots are the mean observed pairwise D_{EST}[2] of each population against all other populations, and blue dots are the mean expected pairwise D_{EST} if alleles are allocated to STs at random (see Lighten et al. for description of randomization protocol; error bars are SDs of 1000 randomizations). See Supplementary Fig. 2 for an enlarged version of the top-left "All STs" plot that includes population names. That the majority of red dots are substantially lower than blue dots shows that ST-based population structure is lower than that expected from marker diversity, suggesting stabilizing selection. This pattern disappears, and may even reverse, if ST9 is deleted, but holds for deletion of each of the other 14 STs. To remove an ST, we removed all alleles from the dataset that were allocated to the focal ST by Lighten et al.[1] Individual fish were only removed from jack-knifed datasets if all their alleles belonged to the focal ST. For a summary of how this affected sample sizes, as well as summary statistics that compare each jack-knifed analysis to the full dataset, see Supplementary Table 1.

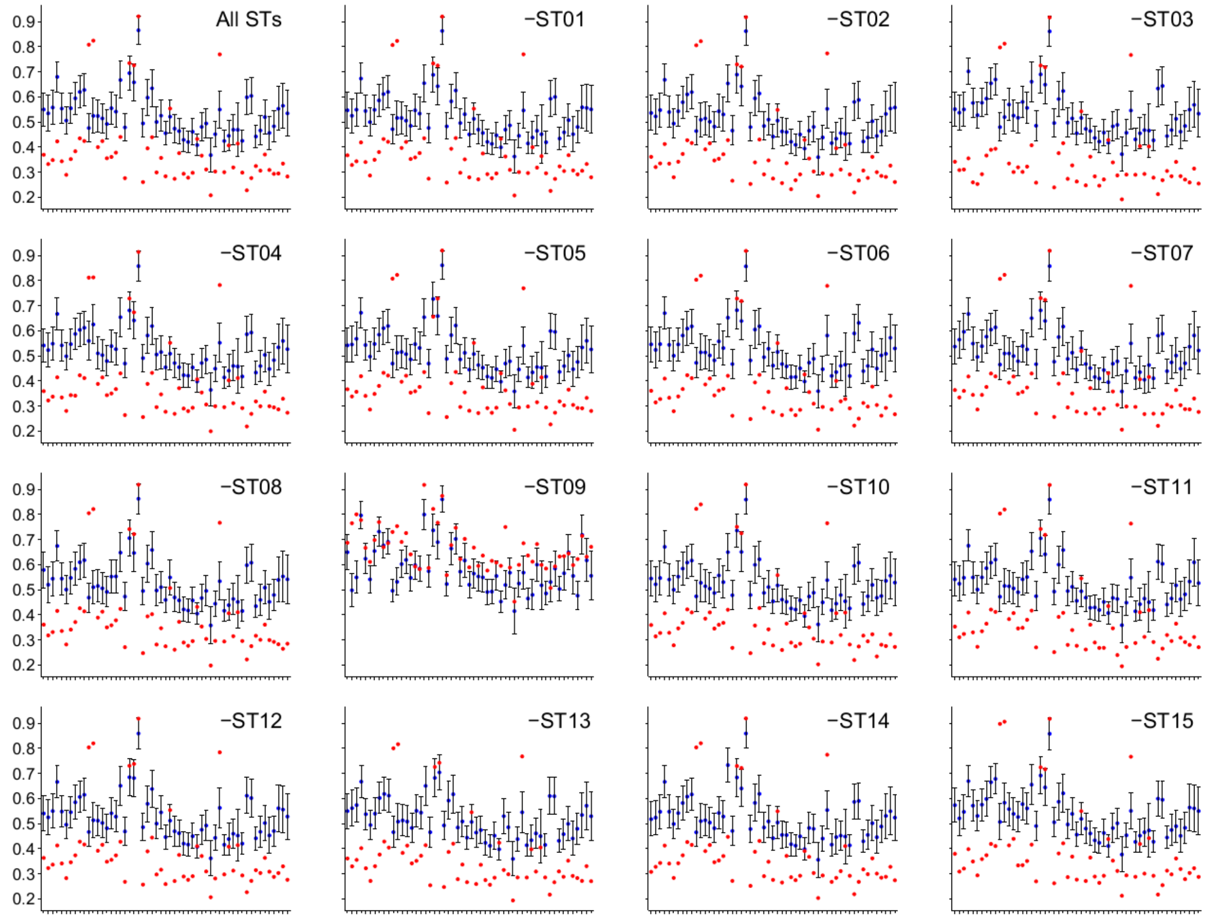

Supplementary Figure 2. Magnified version of the top-left, “All STs” panel of Supplementary Fig. 1, showing population names on the x-axis. Red dots, blue dots, and error bars are as described in the legend to Supplementary Fig. 1.

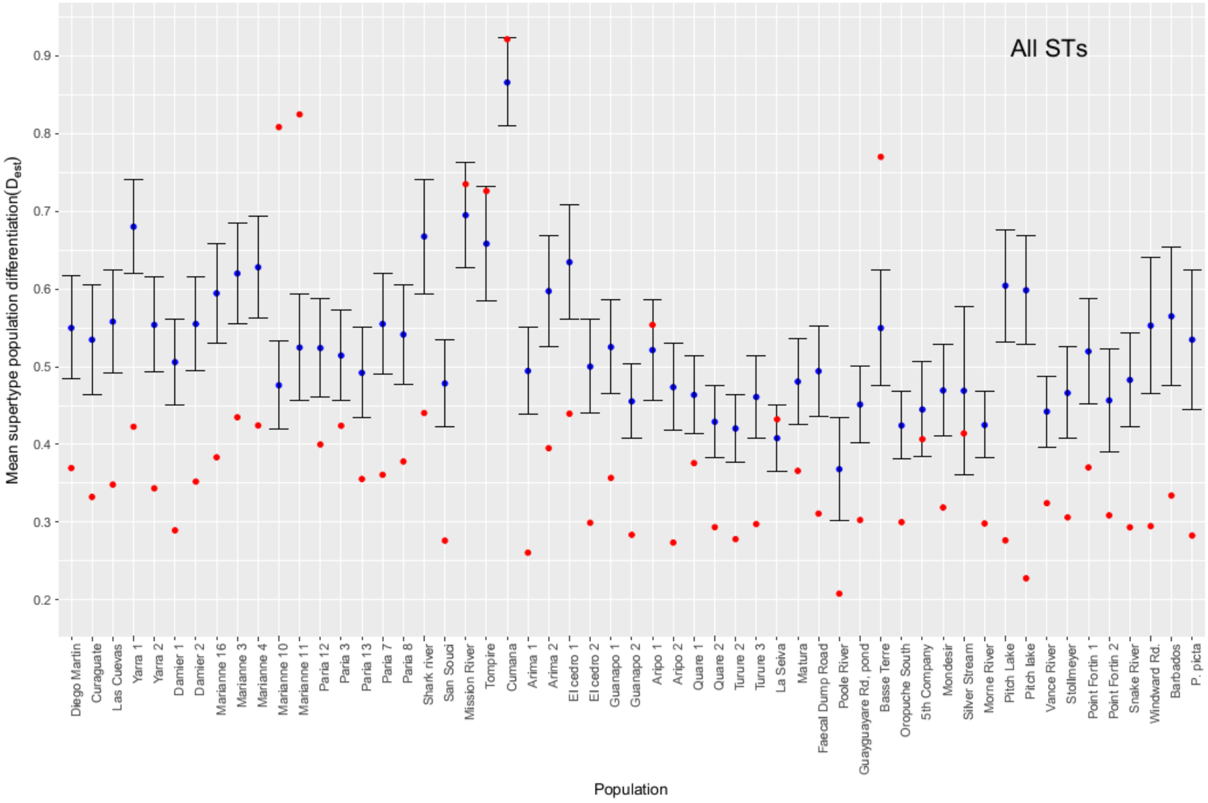

Supplementary Figure 3. Results of simulations in MatLab replicating the model of Lighten et al., using the same parameters and retrieving the same result (i.e. temporal stability of supertypes in physicochemical parameter space) as the original study. Parasite haplotypes (yellow) and host supertypes (black and other colors) are represented as coordinates in 1000 x 1000 grid, reflecting their functional properties (the closer a parasite is to the host, the more likely is a successful host immune response). The parameters are the same as those Lighten et al. used to produce their Fig.4 (N=10 000, u=0.1, 1% random parasite genotypes seeded each generation). Effective number of alleles (#alleles) has been calculated for a sample of 100 individuals.

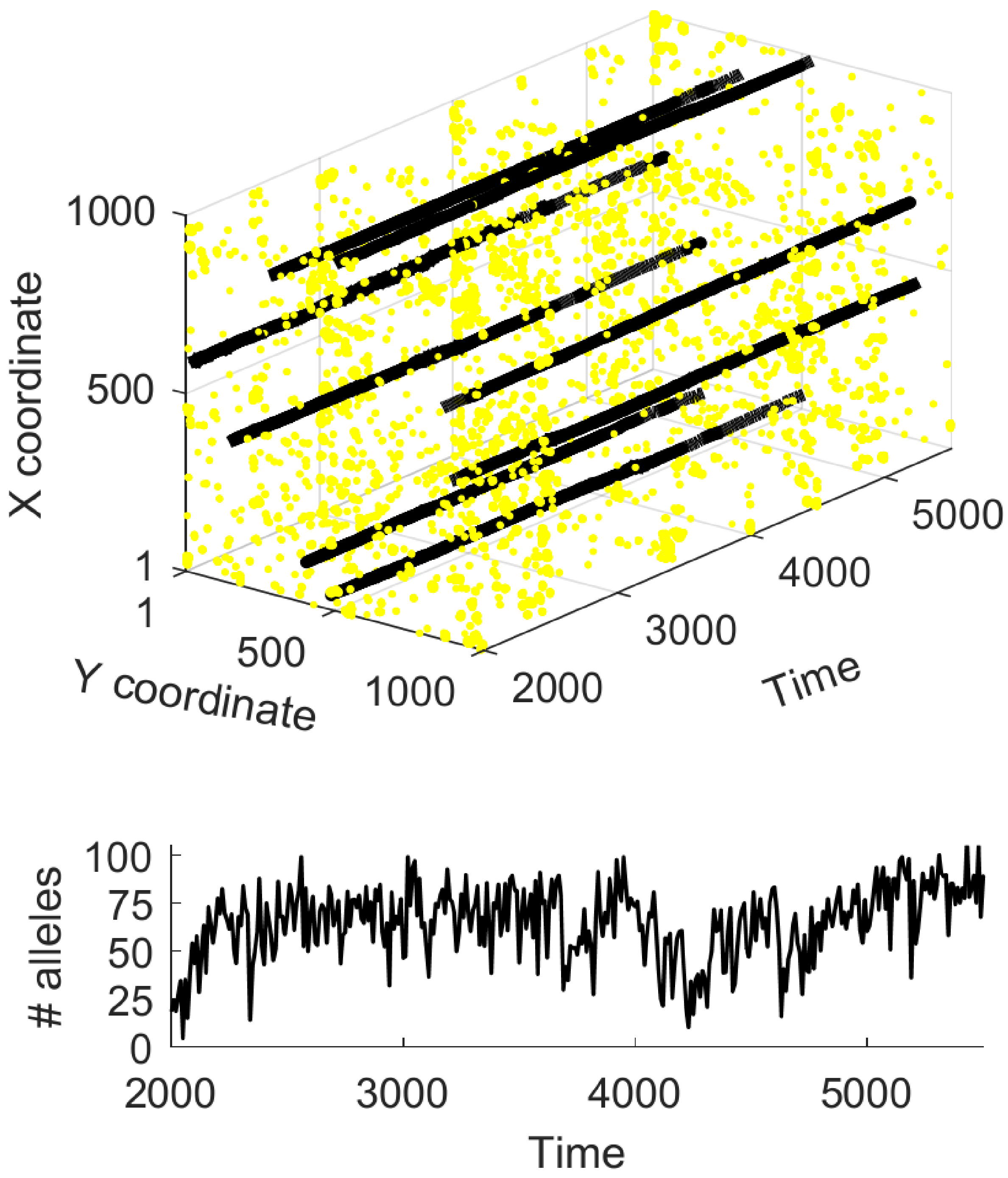

Supplementary Figure 4. Extension of simulations presented in Fig 2D to more parasite species. Three independent runs are shown for each number of parasites. Parasite haplotypes (yellow) and host supertypes (other colors) are represented as coordinates in 1000 x 1000 grid, reflecting their functional properties (the closer a parasite is to the host, the more likely is a successful host immune response). The parameters used here are the same as those Lighten et al. used to produce their Fig.4 (N=10 000, u=0.1) but with no random pathogen genotypes seeded. Effective number of alleles (#alleles) has been calculated for a sample of 100 individuals. The results are qualitatively consistent for different numbers of parasites: multiple supertypes and large numbers of alleles are retained in the host population, but supertype positions in physicochemical parameter space are not temporally stable.

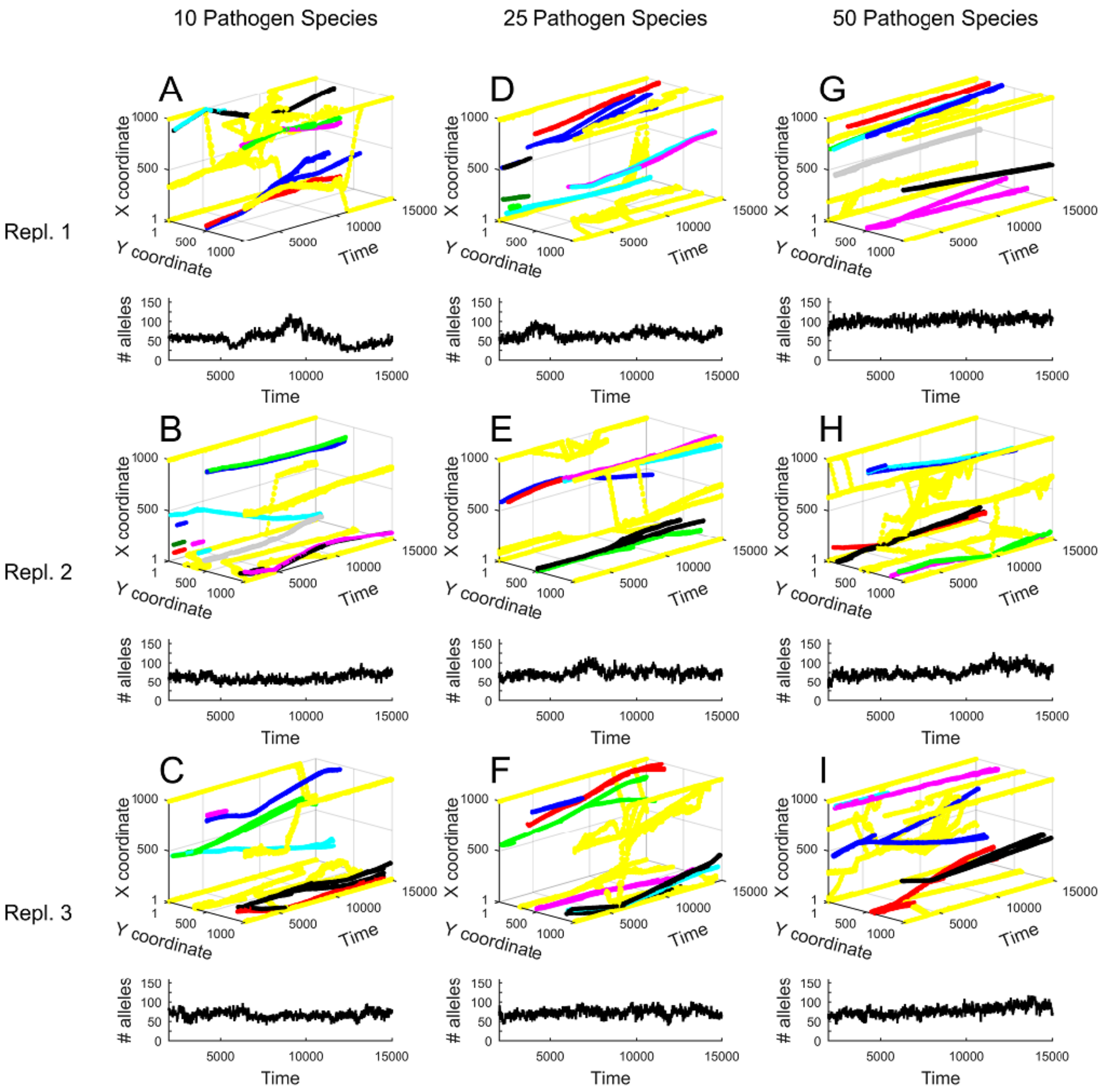

Supplementary Figure 5. Simulations using 10 independent parasites, with all parameters as in Fig. 2D (no random parasite genotypes seeded) except that mutation rates were decreased to 10^{-2} for pathogens and 10^{-3} for hosts. Three independent runs are shown for each population size. Parasite haplotypes (yellow) and host supertypes (other colors) are represented as coordinates in 1000 x 1000 grid, reflecting their functional properties (the closer a parasite is to the host, the more likely is a successful host immune response). Upper row A-C: 10 000 hosts, lower row D-F: 100 000 hosts. Effective number of alleles (#alleles) has been calculated for a sample of 100 individuals. With these mutation rates, the effective number of alleles is lower much than that routinely seen in wild populations.

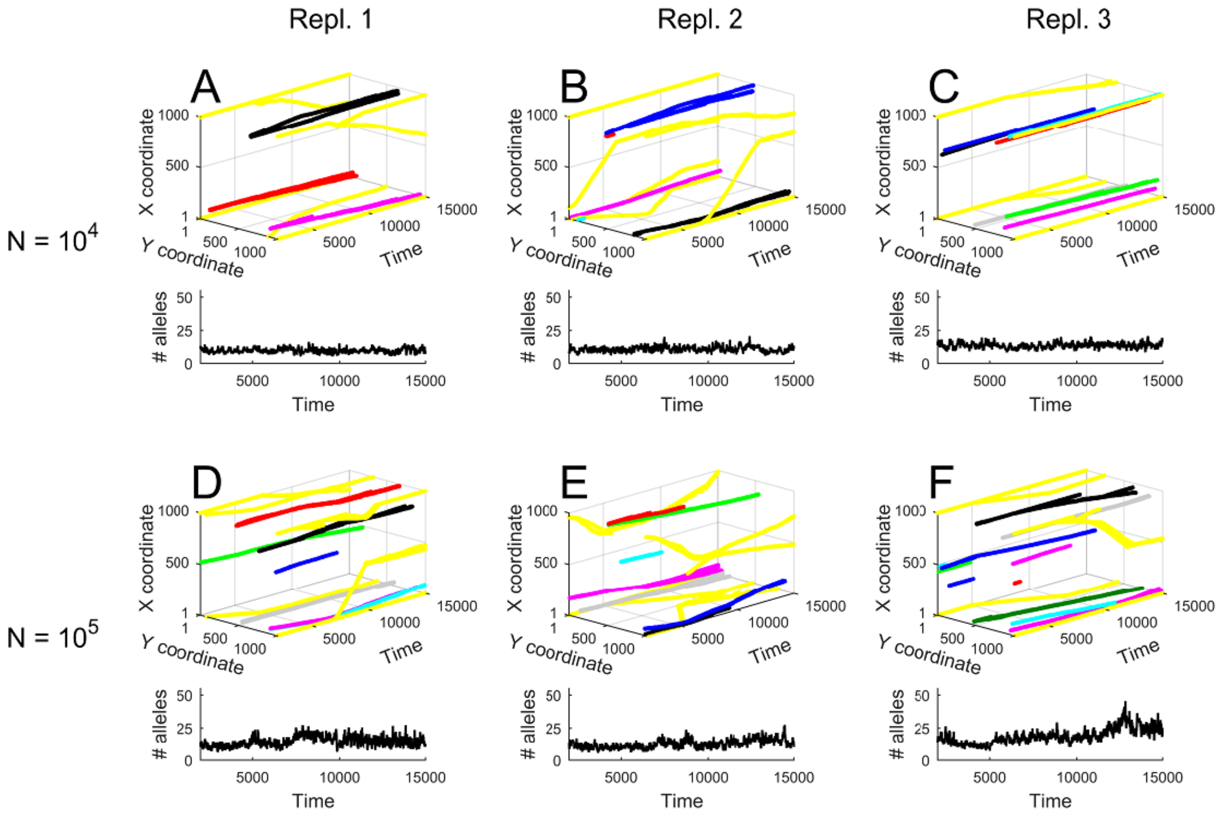

Supplementary Figure 6. Results of simulations that do not seed each pathogen generation with ≈100 new genotypes, as in Fig. 2C, and with selection on pathogens “turned off”, as in Fig. 2B. (A-C are three independent runs. Parasite haplotypes (yellow) and host supertypes (other colors) are represented as coordinates in 1000 x 1000 grid (the distance between a parasite and the host, determine fitness of hosts but not parasites). Supertype diversity quickly declines.

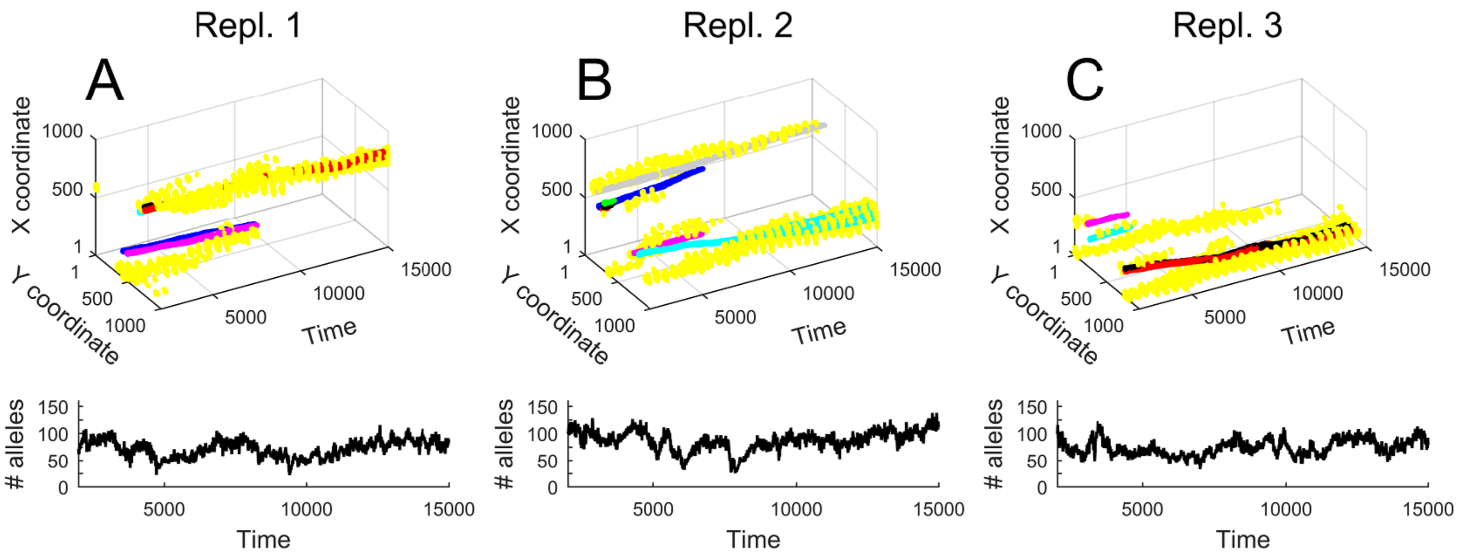

Supplementary Table 1. Summary statistics for each jack-knifed dataset (delete-one supertype; ST) shown in Supplementary Fig. 1. “Mean obs.-exp. diff. (± SD)”: for deletion of each ST, mean difference between observed pairwise population genetic structure by STs (red dots of each panel in Fig. S1) and structure expected if alleles are assigned to STs at random *pro rata* (blue dots). Values are D_{EST}^2, and means and SDs are unweighted. “r_s obs. v. full data”: correlation coefficient (Spearman) of each jack-knifed dataset’s red dots against the red dots of the full dataset. The higher the r_s value, the more similar a jack-knifed dataset is to the full dataset in terms of rank order of red dots. Together, these metrics suggest that ST9 exerts a disproportionate effect on the observed population structure and its interpretation. “Allele freq.” is the combined frequency of the alleles in the focal ST, expressed as number of alleles rather than number of carriers. “N. fish dropped” is the number of fish (out of the total of 1586) removed from each jack-knifed dataset for having only alleles in the focal ST. One population (Cumana; n = 6) was fixed for ST13 (§) and was thus dropped from this jack-knifed dataset. We performed three different resampling approaches (Resamp. 1 – 3) to investigate whether the impact of removing a given ST (measured by “r_s obs. v. full data” – generating blue dots for each resample would be too computationally intensive) can be explained solely by the number of unique nucleotide sequences that the ST contains, or by the frequency of alleles representing the ST in the full dataset; if such were the case, for an ST with n instances or n amino acid sequences, removing n random sequences or instances, irrespective of their ST, should result in a similar r_S to that reported in the “r_s obs. v. full data” column. “Quantile” is the position of the observed value on the respective distribution of bootstrapped values. *P*-values are two-tailed bootstrap *P*-value for the hypothesis that the observed r_s = mean bootstrap r_s. Resamp. 1 assesses the effect of the number of unique nucleotide sequences in a given ST. For each ST, it repeatedly removes all occurrences of a random subset of sequences from the dataset, corresponding in size to the number of sequences observed in that ST. Resamp. 2 makes deletions that match the frequency of the focal ST but without systematically removing any particular sequences. Resamp. 3 is a spatially structured version of Resamp. 2, and matches the number of random deletions within each population to the frequency of the focal ST within each population. The influence of ST9 is much greater than one would expect for all three resampling approaches. No other ST is significant for all three approaches.

ST	Mean obs.-exp. diff. (± SD)	r_s obs. v. full data	N. alleles in ST	ST allele freq.	N. fish dropped	Resamp. 1		Resamp. 2		Resamp. 3	
						Mean r_s	Quantile	Mean r_s	Quantile	Mean r_s	Quantile
1	-0.13 ± 0.13	>0.99	16	67	0	0.97	0.888	>0.99	0.306	0.98	0.906
2	-0.13 ± 0.14	0.99	25	156	4	0.95	0.795	0.99	0.201	>0.99	0.189
3	-0.17 ± 0.15	0.96	32	553	13	0.94	0.567	0.97	0.177	0.99	**0.002
4	-0.14 ± 0.12	>0.99	38	248	18	0.93	**0.999	0.99	0.963	>0.99	0.222
5	-0.13 ± 0.13	>0.99	21	79	8	0.96	**0.998	>0.99	0.890	>0.99	0.616
6	-0.14 ± 0.14	0.99	44	221	9	0.91	0.979	0.99	0.350	0.99	0.876

7	-0.13 ± 0.13	0.98	35	142	3	0.93	0.837	>0.99	†0.039	0.99	0.319
8	-0.14 ± 0.14	0.98	32	225	0	0.94	0.760	0.99	0.093	0.98	0.225
9	0.08 ± 0.07	0.67	55	1799	14	0.90	*0.014	0.91	***<0.000	0.91	***<0.000
10	-0.14 ± 0.14	0.98	48	252	11	0.91	0.955	0.99	0.195	0.98	0.308
11	-0.14 ± 0.14	0.98	21	305	6	0.96	0.454	0.98	0.144	0.97	0.506
12	-0.13 ± 0.13	>0.99	38	220	3	0.93	*0.992	0.99	0.798	>0.99	0.470
13*	-0.15 ± 0.14	>0.99	49	248	§10	0.91	***>0.999	0.99	0.822	0.99	0.743
14	-0.13 ± 0.13	0.96	35	147	1	0.93	0.561	0.99	*0.007	>0.99	***<0.000
15	-0.14 ± 0.14	0.98	50	492	7	0.91	0.957	0.97	0.790	0.98	0.739

P-values: † < 0.10; * < 0.05; ** < 0.01; *** < 0.001

Supplementary References

1 Lighten, J. *et al.* Evolutionary genetics of immunological supertypes reveals two faces of the Red Queen. *Nature Communications* 8, 1294, doi:10.1038/s41467-017-01183-2 (2017).

2 Jost, L. G<inf>ST</inf> and its relatives do not measure differentiation. *Molecular Ecology* 17, 4015-4026, doi:10.1111/j.1365-294X.2008.03887.x (2008).
