# Supplementary material for: The role of MHC supertypes in promoting trans-species polymorphism remains an open question
Source: Nat Commun. 2018 Oct 19;9:4362. doi: 10.1038/s41467-018-06821-x (PMC6195607; doi:10.1038/s41467-018-06821-x)
Supplement: Supplementary file 3 — Description of Additional Supplementary Files [file 41467_2018_6821_MOESM3_ESM.pdf]

Description of Additional Supplementary Files

File Name: Supplementary Data 1
Description: The MatLab code used to run simulations.
